# The DNA Methylome and Transcriptome of Different Brain Regions in Schizophrenia and Bipolar Disorder

**DOI:** 10.1371/journal.pone.0095875

**Published:** 2014-04-28

**Authors:** Yun Xiao, Cynthia Camarillo, Yanyan Ping, Tania Bedard Arana, Hongying Zhao, Peter M. Thompson, Chaohan Xu, Bin Brenda Su, Huihui Fan, Javier Ordonez, Li Wang, Chunxiang Mao, Yunpeng Zhang, Dianne Cruz, Michael A. Escamilla, Xia Li, Chun Xu

**Affiliations:** 1 Departments of Psychiatry, Texas Tech University Health Science Center, El Paso, Texas, United States of America; 2 College of Bioinformatics Science and Technology, Harbin Medical University, Harbin, Heilongjiang, China; 3 The Center of Excellence in Neuroscience, Texas Tech University Health Science Center, El Paso, Texas, United States of America; 4 Southwest Brain Bank, Department of Psychiatry, University of Texas Health Science Center at San Antonio, San Antonio, Texas, United States of America; 5 University of Toronto, Toronto, Canada; University of Illinois at Chicago, United States of America

## Abstract

Extensive changes in DNA methylation have been observed in schizophrenia (SC) and bipolar disorder (BP), and may contribute to the pathogenesis of these disorders. Here, we performed genome-scale DNA methylation profiling using methylated DNA immunoprecipitation followed by sequencing (MeDIP-seq) on two brain regions (including frontal cortex and anterior cingulate) in 5 SC, 7 BP and 6 normal subjects. Comparing with normal controls, we identified substantial differentially methylated regions (DMRs) in these two brain regions of SC and BP. To our surprise, different brain regions show completely distinct distributions of DMRs across the genomes. In frontal cortex of both SC and BP subjects, we observed widespread hypomethylation as compared to normal controls, preferentially targeting the terminal ends of the chromosomes. In contrast, in anterior cingulate, both SC and BP subjects displayed extensive gain of methylation. Notably, in these two brain regions of SC and BP, only a few DMRs overlapped with promoters, whereas a greater proportion occurs in introns and intergenic regions. Functional enrichment analysis indicated that important psychiatric disorder-related biological processes such as neuron development, differentiation and projection may be altered by epigenetic changes located in the intronic regions. Transcriptome analysis revealed consistent dysfunctional processes with those determined by DMRs. Furthermore, DMRs in the same brain regions from SC and BP could successfully distinguish BP and/or SC from normal controls while differentially expressed genes could not. Overall, our results support a major role for brain-region-dependent aberrant DNA methylation in the pathogenesis of these two disorders.

## Introduction

Psychiatric disorders characterized by long-lasting behavioral abnormalities constitute a considerable public health burden [1]. Two major psychiatric disorders including schizophrenia (SC) and bipolar disorder (BP) have received considerable attention in molecular biological studies; nevertheless their etiology remains largely enigmatic. Despite the completion of numerous large-scale genome-wide association studies and the recent application of exon sequencing to identify risk loci and structural genomic variants (e.g. copy number variation) associated with these psychiatric disorders, it is becoming clear that the few number of risk genes/loci and extremely rare structural variants are insufficient to account for the risk of psychiatric disorders [2]. This is because most psychiatric disorders are associated with molecular abnormalities in multiple genes and signals that control their expression, rather than mere genetic variants in a few genes.

Increasing evidence suggests that epigenetic modification plays important roles in normal biology (e.g. development) and disease (e.g. psychiatric disorders) by influencing gene expression. As one type of epigenetic events, DNA methylation has been extensively explored in different cellular conditions [3]–[6], whose abnormalities at specific regions can induce expression changes mostly through alterations of chromosomal accessibility or local chromatin structure. There is mounting evidence that DNA methylation is involved in the pathogenesis of SC and BP. Initial studies focused on DNA methylation alterations in candidate genes, such as *RELN* [7], [8], *COMT* [9] and *GAD67* [10]. The first epigenome-wide study performed by Mill *et al*. [11] comprehensively characterized DNA methylation in the prefrontal cortex of patients with major psychosis by investigating ∼27,000 CpG dinucleotides using microarray. They identified significant epigenetic changes associated with SC and BP. Subsequently, Dempster *et al*. [12] performed genome-wide analysis of DNA methylation of blood samples from 22 twin pairs discordant for SC and BP using microarray and further demonstrated important DNA methylation changes in the molecular mechanisms associated with SC and BP.

Here, we performed a genome-wide DNA methylation analysis in two brain regions, Brodmann area 9 (BA9, part of the frontal cortex) and Brodmann area 24 (BA24, part of the anterior cingulate) from patients with SC or BP and normal controls using methylated DNA immunoprecipitation and sequencing (MeDIP-seq) [13], which provided more comprehensive DNA methylation interrogation than microarray-based technologies, and then comprehensively characterized DNA methylation alterations in different brain regions of SC and BP. Furthermore, we detected transcriptome-wide gene expression changes between patients and controls using RNA-seq. Finally, we combined DNA methylome and transcriptome to explore their possible links in different brain regions of SC and BP.

## Materials and Methods

### Ethics statement

The study was approved by the institutional review board of Texas Tech University Health Science Center, Texas, United States. All patients provided written informed consent.

### Patient samples

Five SC, seven BP and six normal samples were included in this study. These samples were collected from the Southwest Brain Bank with consent from the next-of-kin (NOK) (see Methods S1 for details). The NOK agreed to provide the donation and they read a State approved form. We called the NOK and recorded their agreement. The NOK interview (psychological autopsy) about the donor was performed by trained clinicians. All of the patients in this study have met best estimate consensus diagnoses of SC or BP as defined by the DSM-IV-TR criteria, as previously reported [14]. These studies have been approved by the Institutional Review Board of the University of Texas Health Science Center at San Antonio. The quality of the postmortem brain tissue was determined by a neuro-pathologist through both gross and microscopic neuropathological examinations. All subjects in this study were free of confounding neuropathology. For tissue identification of BA9 and BA24 taken from the same hemisphere, we used the criteria described by Rajkowska and Goldman-Rakic [15].

### MeDIP-seq

The genomic DNA was extracted from two brain regions (BA9 and BA24) in samples as detailed in the Methods S1. Solexa libraries were subsequently prepared as follows: at least 5 µg genome DNA was fragmented to a mean size of approximately 250 bp by sonication, followed by the blunt-ending and dA addition to the 3′-end. Adapters were then ligated to the end of DNA fragments. Double-stranded DNA was denatured and the DNA fragments were immunoprecipitated by 5 mC antibodies. Real-time PCR was used to validate the quality of immunoprecipitation. After PCR amplification, the material was sequenced using the genome-wide massively parallel paired-end sequencing platform Illumina HiSeq2000 (read length of 2×50 bp).

### Identification of differentially methylated regions

After removing low-quality reads (reads containing Ns>5) using a custom script, we aligned reads to the standard hg19 build of the human reference genome using SOAP (version 2.20) allowing up to two mismatches [16]. Only uniquely mapped reads were used for further analysis. Whole genome peak scanning was performed by MACS (with default parameters, version 1.4.2) that models the number of reads from a genomic region as a Poisson distribution [17]. A genomic region with a p value<10e-5 was defined as a significant peak. For each peak on autosomes, using a custom script, we calculated reads per kilobase per million mapped reads (RPKM) for each significant peak in a specific sample as its DNA methylation level 
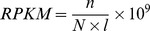
where *n* represents read counts within a peak, *N* represents total read counts and *l* represents the length of the peak. The two-sided t-test was used to identify significantly differentially methylated peaks with p value<0.01. Then, two significant peaks were merged if their spacing was less than 50 bp. The merged significant peaks were regarded as differentially methylated regions (DMRs).

In order to identify DMR-related genes, we obtained known gene location information from UCSC known gene track (hg19). The genes with at least one element (including promoter, 5′UTR, exon, intron and 3′UTR) overlapping with DMRs were selected for functional characterization. Regions from −2.5 kb upstream to +0.5 kb downstream of transcriptional start sites were defined as promoters of genes.

### RNA-seq

A detailed description of RNA extraction methods as well as the entire experimental setup has been published previously [18]. Briefly, the tissue samples were homogenized in TRIzol solvent (Invitrogen, Carlsbad, CA, USA), and the total RNA was isolated with RNeasy Lipid Tissue Mini Kit (Qiagen # 74804) and QIAzol Lysis Reagent (Qiagen # 79306). Five hundred nanogram RNA was reverse-transcribed to cDNA which was used as template in TaqMan Gene Expression Assays (Applied Biosystems). The quality of postmortem brain tissue was determined by toxicology studies, brain tissue pH [19], [20] and RNA integrity number [21]. The postmortem interval was limited to 36 h (ranges between 13–36 h). Beads with oligo(dT) were used to isolate poly(A) mRNA after total RNA was collected from all samples. Fragmentation buffer was added to interrupt mRNA to fragments. Taking these 200∼300 bp fragments as templates, random hexamer-primer was used to synthesize the first-strand cDNA. The second-strand cDNA was synthesized using buffer, dNTPs, RNase H and DNA polymerase I. Fragments were purified with QiaQuick PCR extraction kit and resolved with EB buffer for end reparation and adding poly(A). The fragments were then connected with sequencing adaptors. Amplification with PCR was done by selecting suitable fragments as templates based on the results of agarose gel electrophoresis. The library was then sequenced as paired-end 90 bp sequence reads using Illumina HiSeq 2000. Reads after low quality filtering were mapped to reference genome (hg19) using SOAP. Mismatches of no more than 2 bases were allowed. The RPKM method [22] was then used to calculate gene expression. Differentially expressed genes were identified with fold changes greater than 1.5. We also applied Cuffdiff (version 2.1.1, with default parameters) [23] to RNA-seq data aligned by TopHat (version 2.0.8, with default parameters) [24] for identification of differentially expressed genes (FDR<0.05).

### Functional enrichments

The hypergeometric distribution test was used to identify biological processes (BP) from Gene Ontology [25] significantly enriched in a specified gene set with FDR<0.05, which was implemented in the Bioconductor software GOstats [26] package together with GO.db package [27]. Because standard hypergeometric test can introduce bias when applied to RPKM transformed RNA-seq data, a bias corrected tool GOseq (a Bioconductor package) were also used to re-perform the function enrichment analysis with the same threshold FDR<0.05 [28].

### Motif discovery in DMRs

The 8–20 nt sequence motifs significantly enriched in DMRs were detected by the software suite HOMER (Hypergeometric Optimization of Motif EnRichment, http://biowhat.ucsd.edu/homer/) [29]. The findMotifsGenome.pl command was run in HOMER using hg19 as the reference genome with default parameters.

We have deposited our dataset (all MeDIP-seq and RNA-seq data) in NIH Short Read Archive (ID: SRP035524).

## Results

### Identification of differentially methylated regions

We detected DNA methylation levels for two human brain regions (BA9 and BA24) from 18 individuals composed of five SC, seven BP and six control subjects (Table S1) by MeDIP-seq that uses antibody-based immunoprecipitation of 5-methylcytosine followed by sequencing the immunoprecipitated fractions. No significant difference was detected between the age of the patients and normal individuals. On average, 73,469,388 paired-end reads were generated for each sample, 87% of which were uniquely aligned to the human genome. We analyzed the distributions of reads around CpG islands (CGI), and observed higher DNA methylation levels at upstream and downstream of CGI (Figure S1A). We further analyzed the distributions of reads around gene body, and found depletion of DNA methylation around transcription start sites (Figure S1B). Subsequently, we divided the genome into 10 kb windows and calculated read per million values for each window. Notably, in many chromosomes, some local methylation changes in the disorders relative to normal controls were found in the BA9 brain region and interestingly, these local changes tended to occur at the terminal ends of the autosomes (Figure S1C and S1D, and Figure S2). In order to characterize the similarities of DNA methylation for each intra-class category, we calculated the methylation levels within 10 kb windows scanned across genome and their pairwise Pearson correlation coefficients. Our results showed consistent methylation patterns (Pearson correlation coefficients from 0.89 to 0.98, with an average of 0.95, Figure S3A). Moreover, we completed the principal component analysis of methylation levels of 10 kb windows for both case and control samples (36 samples in total) and did not found obvious outliers (Figure S4A).

**Figure 1:**
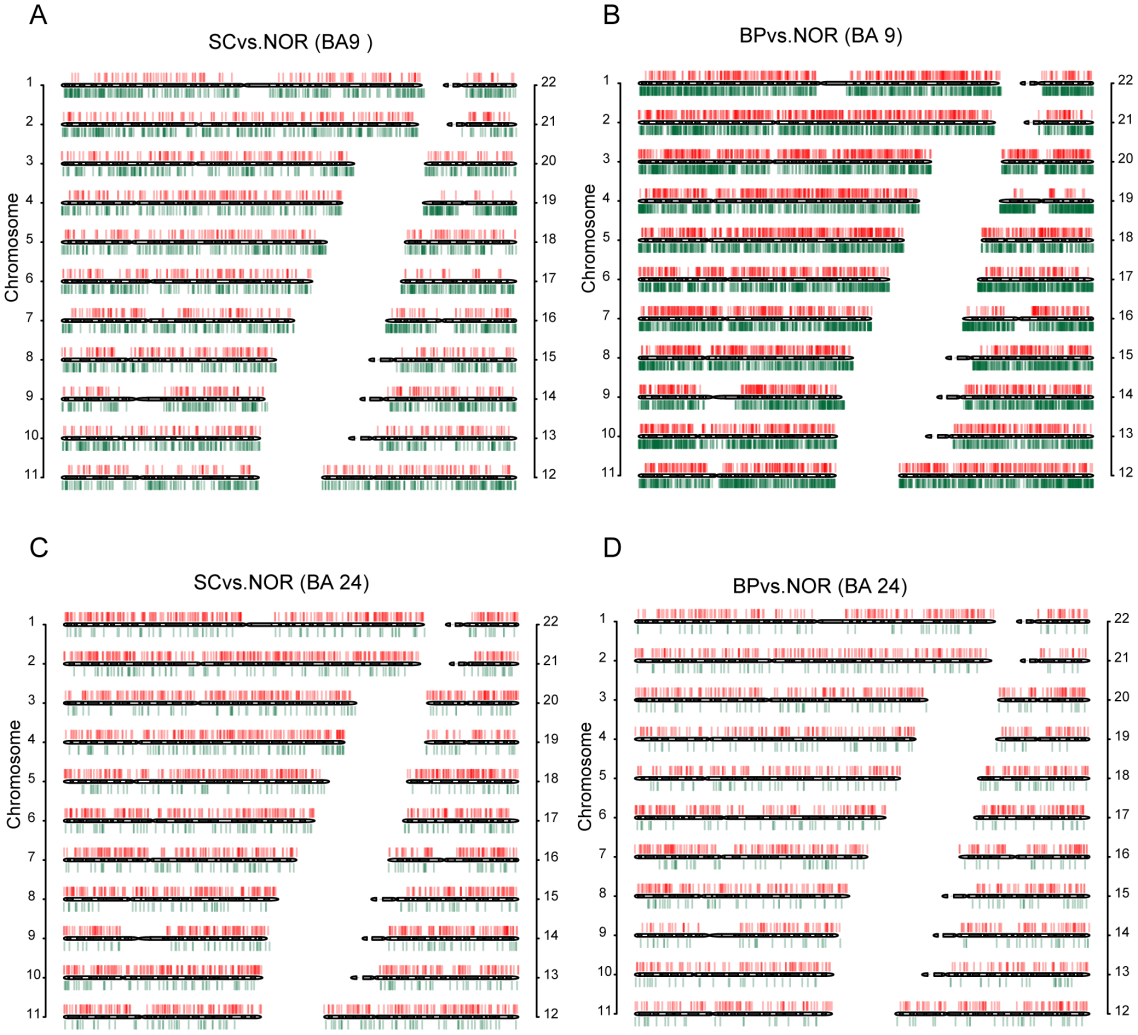
The distribution of hyper- and hypomethylated DMRs. Autosome ideogram representing differential methylation in the BA9 brain regions of SC vs. normal (A), BP vs. normal (B), and in the BA24 brain regions of SC vs. normal (C) and BP vs. normal (D). Red points represent hypermethylation and green ones represent hypomethylation relative to normal subjects.

**Figure 2:**
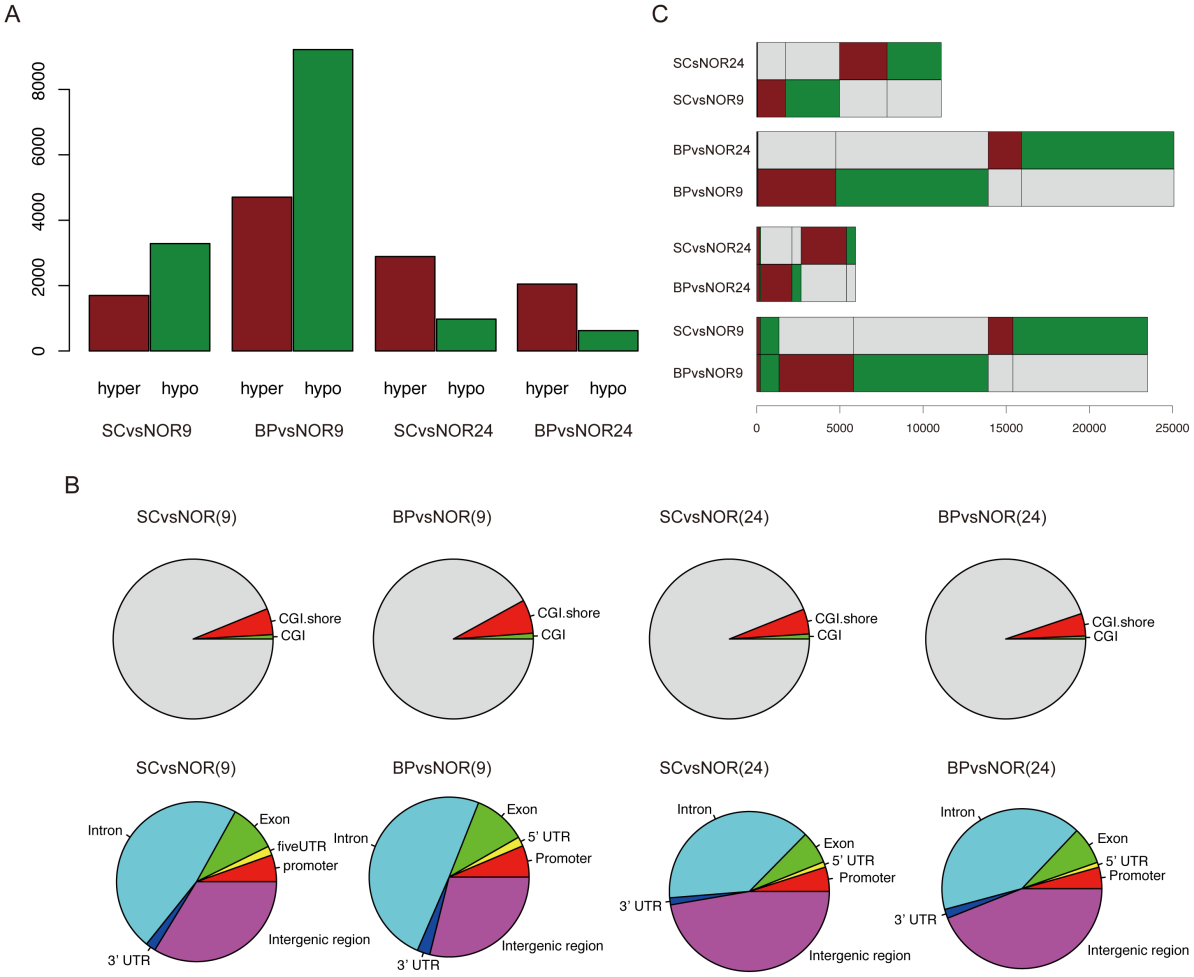
Features of DMRs. (A) DMRs in distinct brain regions of SC and BP. (B) DNA methylation alteration patterns across CGIs and gene elements. (C) Overlapping of DMRs between different brain regions of SC and BP. Red color represents hypermethylation and green color represents hypomethylation. Gray color represents that DMRs in one comparison do not overlap with hyper- or hypomethylated DMRs in the other comparison.

Whole genome peak scanning was then carried out using MACS [30], [31]. Using the RPKM method, we calculated DNA methylation levels for peaks and then identified differentially methylated regions (DMRs) between disease (SC or BP) and normal subjects. We identified 4985 and 13925 DMRs in the BA9 region of SC and BP samples, respectively, and 3867 and 2672 DMRs in the BA24 regions of SC and BP samples, respectively, in comparison to the corresponding normal samples (Table S2). DMRs in the two brain regions of SC and BP have similar lengths, with an average length of 1.2 kb. A much higher number of DMRs in the BA9 regions of SC and BP was observed than those in the BA24 regions. The highest number of DMRs was observed in the BA9 region of BP samples. To our surprise, both SC and BP showed different DNA methylation alternation patterns between the two brain regions. In the BA9 regions of SC and BP, most autosomes showed obvious asymmetrical distributions of hyper- and hypomethylations, that was, a predominance of hypomethylation present in these autosomes, with a few dispersed hypermethylated DMRs (Figure 1A and 1B). In contrast, profound hypermethylation distributed across all autosomes was identified in the BA24 regions of SC and BP subjects (Figure 1C and 1D). More hypomethylated DMRs in the BA9 regions of SC and BP samples, and more hypermethylated DMRs in the BA24 regions were observed (Figure 2A). Furthermore, we also used limma [32], [33] to detect DMRs between case and control adjusting for gender and age. With a threshold of FDR 0.05, we did not find any significant DMRs. Instead, using p value of 0.05, we identified 17 hyper- and 621 hypomethylated DMRs of SC and 213 hyper- and 1253 hypomethylated ones of BP in the BA9 regions, and 620 hyper- and 29 hypomethylated DMRs of SC and 136 hyper- and 77 hypomethylated DMRs of BP in the BA24 regions, which showed similar DMR patterns as previous ones. Our findings suggest that DNA methylation alterations in these two disorders may be heavily dependent on distinct brain regions.

Through examination of the methylation alterations around CGI (CGI and CGI shores) in SC and BP samples, we found that more DMRs in the BA9 and BA24 overlap with CGI shores relative to CGI (odd ratio = 0.16, p value = 4.79e-141 for BP in BA9; odd ratio = 0.16, p value = 3.12e-40 for SC in BA9; odd ratio = 0.13, p value = 7.60e-22 for BP in BA24; odd ratio = 0.19, p value = 7.71e-27 for SC in BA24; Fisher's exact test), consistent with previous findings in other diseases [34], whereas the majority of DMRs were located outside the CGIs and their shores (Figure 2B). Moreover, the DNA methylation alteration patterns were consistent across different brain regions of SC and BP subjects, suggesting that the preference of DNA methylation alternations at CGI shores may be an inherent nature irrespective of brain regions and disease types. We also examined the distribution of DMRs across different gene elements including promoter, 5′UTR, exon, intron, 3′UTR and intergenic region (Figure 2B). There were only parts of DMRs overlapping with promoters, which was in line with recent observations in many genome-wide DNA methylation studies [35]. To determine significant difference in the distribution of DMRs in various elements, we performed a permutation analysis. In details, a set of random regions was randomly generated from genomes, keeping the same distribution of chromosome, length and size as real DMRs. The set of random regions was defined as a pseudo-random DMR set. This process was then repeated 1000 times to generate 1000 pseudo-random DMR sets in each comparison between patients (BP or SC) and normal subjects. For each type of elements, we computed the percentage of pseudo-random DMR sets that showed more overlap with the elements than the real DMR set as the p value for the statistical significance of the enrichment of DMRs in the elements. We found that DMRs in the BA9 region of BP were significantly enriched in various gene elements including promoter, 3′ UTR, intron, exon and 5′ UTR (p value = 0.001). In the same brain region, DMRs in SC were significantly enriched in intron and exon (p value = 0.001 and p value = 0.049, respectively). However, in the BA24 brain region, only the intergenic regions were found to be significantly enriched by DMRs in SC (p value = 0.001). Interestingly, the proportions of DMRs overlapping with introns in the BA9 region of SC and BP samples were substantially higher than those in the BA24 region (odd ratio = 1.45, p value = 9.89e-18 for SC and odd ratio = 1.37, p value = 1.13e-13 for BP, Fisher's exact test). However, the proportions of DMRs overlapping with intergenic regions in the BA9 were obviously lower than those in the BA24 region (odd ratio = 0.66, p value = 7.25e-22 for SC and odd ratio = 0.67, p value = 1.47e-20 for BP, Fisher's exact test).

Also, we investigated DMR-related genes identified in SC and BP. Numerous known SC and BP genes, including *RELN*, *PPP3CC*, *DNMT1*, *DTNBP1*, *NOS1*, *HTR1E*, *GRM5*, *PRIMA1*, *HTR2A* and *HTR2A*, were found. *RELN* is one of the most abnormal markers in the context of SC and BP [36]. MRNA and protein expression levels of *RELN* have been observed to be severely reduced in various cortical structures of postmortem brain from SC and BP [37], [38] with its promoter hypermethylated [7]. In addition, the mRNA encoding the DNA methyltransferase enzyme, *DNMT1*, is up-regulated in the neurons accompanied with reduced expression of *RELN* [39]. Also, we compared our findings with gene lists identified in the study of (Mill et al., 2008) and found 57 common genes. One of these common genes, the dystrobrevin binding protein 1 (*DTNBP1*), has been found to harbor a potential susceptibility locus for SC. A recent study also demonstrated that *DTNBP1* encoding a susceptibility protein in SC was important for AMPAR-mediated synaptic transmission and plasticity in the developing hippocampus [40].

Further, we compared DMRs from different brain regions of the same disease. Strikingly, only a few overlapping DMRs between different brain regions were found in the same disease (Figure 2C). There were only 25 hyper- and 20 hypomethylated DMRs in the BA9 region of BP subjects overlapping with hyper- and hypomethylated DMRs in the BA24 region of BP, respectively. Three genes including *COL1A2*, *LMO1* and *IGDCC4* located in the common hyper-DMRs in BP across the two brain regions, without significantly differential expression. Only one gene (*hsa-mir-4266*) was found to be located in the common hyper-DMRs in SC. By comparison, more overlapping DMRs between these two disorders from the same brain regions were found. We found 220 hyper- and 1123 hypomethylated DMRs in the BA9 region of BP samples overlapping with DMRs in the same brain region of SC samples, but without statistical significance (hypergeometric test). These are six genes for hyper-DMRs and 86 genes for hypo-DMRs between BP and SC in the BA9 region. Among the 86 genes, *DNMT1* has already been reported to be associated with SC [39], 15 genes were confirmed by a previous study of Weber et al. [13], and 5 genes showed different expression of FC larger than 1.5. Our results suggest that different brain regions exhibit completely different DNA methylation alternations even within the same disease, whereas some shared dysfunctions of DNA methylation occur in the same brain regions of these two disorders.

### DMR-related functions

**Figure 3:**
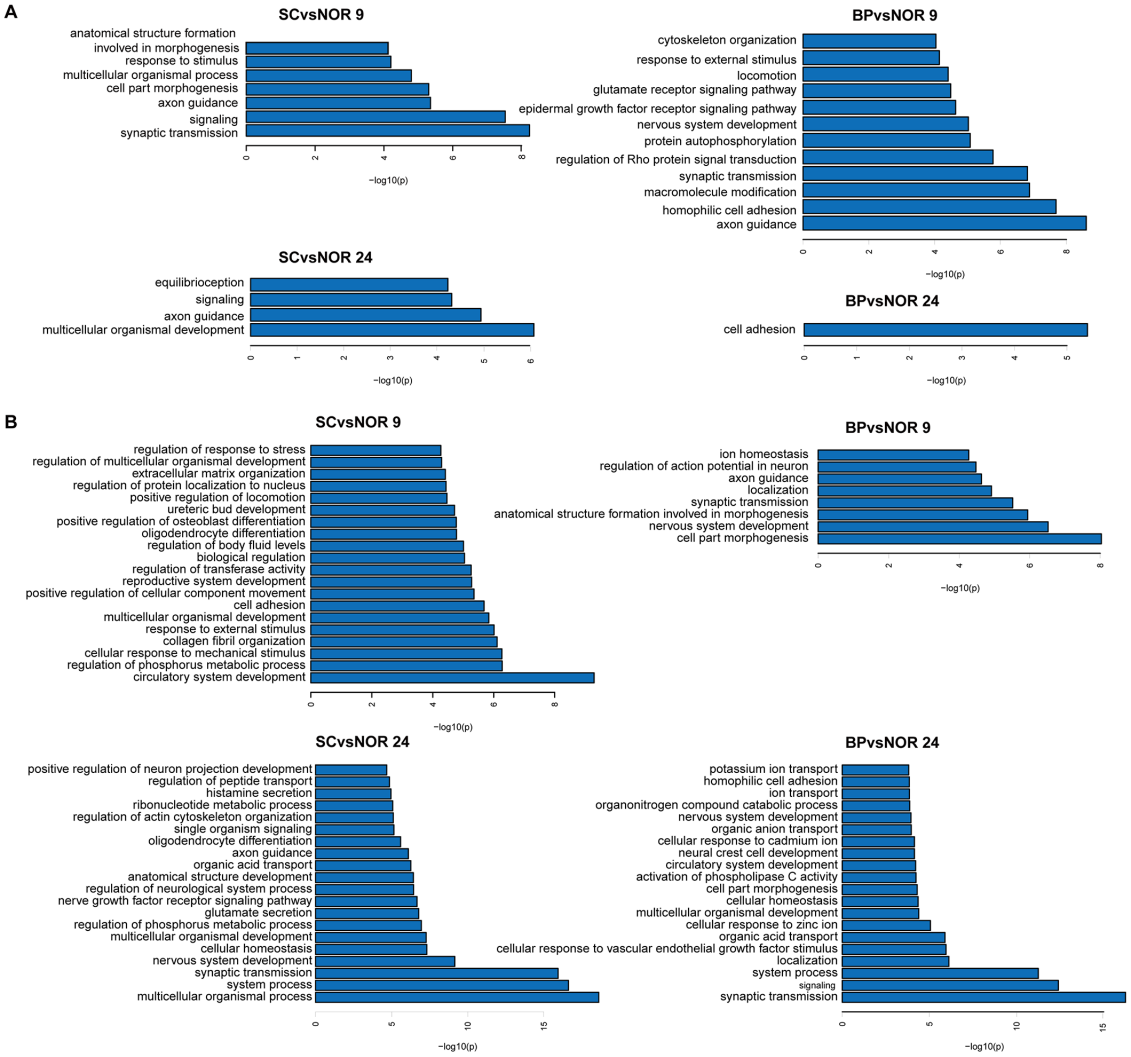
Functional enrichment analyses using DMR-related genes and differentially expressed genes. (A) The top 20 biological processes determined by functional enrichment analyses of DMR-related genes. (B) The top 20 biological processes determined by functional enrichment analyses of significantly differentially expressed genes.

**Figure 4:**
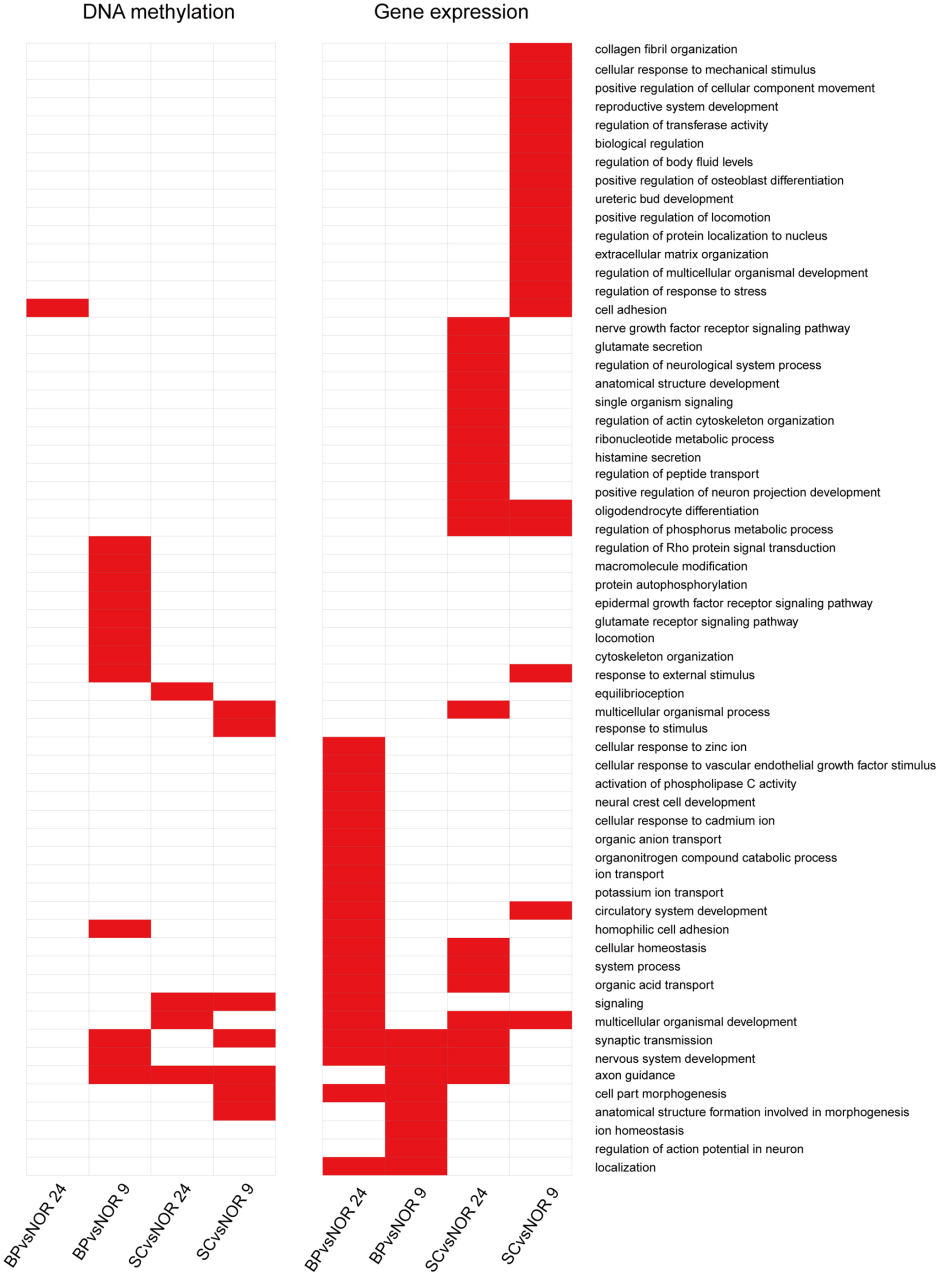
Comparisons of biological processes between different brain regions of SC and BP. The significant biological processes were determined based on DNA methylation alternation (left) and transcriptional changes (right).

Through function enrichment analyses based on DMR-related genes (960, 4497, 1268 and 1955 in the BA9 and BA24 regions of BP and SC respectively), we found the over-representation of many brain-related biological processes (Figure 3A and Table S3), such as neuron development and axon guidance, consistent with previous reports [11]. Notably, many common biological processes were identified between different comparison groups (Figure 4). For example, two common biological processes between BA9 and BA24 brain regions of SC were identified, although they showed a few overlapping DMRs. In particular, axon guidance and signaling were observed in all comparisons except for the BA24 region of BP. Multicellular organismal development was observed in all comparisons of SC. Interestingly, nervous system development was found to be only present in the BA9 region of BP, and only one biological process ‘cell adhesion’ was significantly enriched by DMR-related genes in the BA24 region of BP. Our results suggest that DNA methylation changes can induce dysfunction of neuron development and projection and in turn contribute to the pathogenesis of psychiatric disorders, and different brain regions exhibit different DNA methylation changes but show similar DMR-related biological processes.

In addition, we further identified biological processes significantly enriched by genes with their different elements overlapping with DMRs (Figure S5 and Table S4). Notably, genes with their promoters overlapping with DMRs were not significantly involved in any biological processes in all comparisons. Surprisingly, many brain-related biological processes, such as neuron development, axon guidance and synaptic transmission, were found to be enriched in genes with their introns overlapping with DMRs rather than promoters, suggesting that DNA methylation alterations in introns may exert important roles in the pathogenesis of these two disorders.

In addition, we detected motifs enriched in DMRs by HOMER (Hypergeometric Optimization of Motif EnRichment) with default parameters [29]. Seven and three known motifs were found to be enriched in the DMRs of BP (BA9) and SC (BA9), respectively (Figure S6). And we also found two known motifs enriched in the DMRs of SC (BA24), including *TP53* and *VDR*. Consistently, *TP53* has been demonstrated to be associated with SC in previous studies [41], suggesting that DNA methylation alteration on regulatory elements can influence the binding affinity of regulators and in turn induce the development of disease.

### Transcriptome of SC and BP

Also, we detected the transcriptional profiles of the corresponding brain regions from SC, BP and control samples using RNA-seq. On average, each sample generated more than 10 million high-quality paired-end reads, with more than 85% reads uniquely mapped to the reference genome. Gene expression was calculated using the RPKM method. To determine intra-class correlation using RNA-seq data, we calculated Pearson correlation coefficients between gene expression profiles for each intra-class category. Results showed high intra-class correlations (Pearson correlation coefficients from 0.68 to 0.97, with an average of 0.91, Figure S3B). By principal component analysis of gene expression, we did not find obvious outliers (Figure S4B). Differentially expressed genes in SC and BP were identified with fold changes greater than 1.5. A total of 1077 and 3639 differential genes were found in the BA9 and BA24 regions of SC, respectively, and 2085 and 1643 were identified in the BA9 and BA24 regions of BP, respectively (Table S2 and Table S5).

Besides, differentially expressed genes in SC and BP were identified using Cuffdiff with FDR less than 0.05. A total of 204 and 1503 differential genes were found in the BA9 and BA24 regions of SC, respectively, and 0 and 70 were identified in the BA9 and BA24 regions of BP, respectively. Comparing with BP, fewer differentially expressed genes were observed between SC and controls in both BA9 and BA24 regions, suggesting an important role of subtle dysregulation of genes in schizophrenia parents.

Through functional enrichments of differentially expressed genes, we identified many brain-related biological processes, such as neuron development, axonogenesis and synaptic transmission (Figure 3B and Table S6). The roles of these biological processes have been demonstrated in a number of neuropsychiatric disorders, including BP and SC [42]. Like functional analyses of DMRs, we also found common biological processes associated with SC and BP in both the BA9 and BA24 regions (Figure 4). Synaptic transmission, nervous system development and axon guidance were observed to be shared among almost all of the comparisons. Importantly, numerous abnormal biological processes inferred from DNA methylome were consistent with those inferred from transcriptome (Figure 4). For instance, multicellular organismal development was identified through both DNA methylation changes and transcriptional changes in the BA24 of SC. Both DMR-related and differentially expressed genes were found to be significantly enriched in synaptic transmission and axon guidance. Our results support that DNA methylation alterations play an important role in the pathogenesis of these two severe psychiatric disorders.

Additionally, considering the bias of standard hypergeometric test for function enrichment analysis when applied to genome-wide RNA-seq data, even using the RPKM transformed data, we also used the GOseq, an R package of bias corrected method [28], to re-perform the function enrichment analysis. We found many consistent biological functions between the results of the standard hypergeometric test and the bias corrected method (Table S7), such as nervous system development and synaptic transmission.

### Common molecular mechanisms between SC and BP

Previous studies have demonstrated that SC and BP shared genetic variation [43], [44]. Consistently, we observed similar abnormal functions between SC and BP when compared to controls. To further characterize their common molecular mechanisms, we combined SC and BP samples to identify common DNA methylation alterations in different brain regions. Our results showed that 2650 DMRs and 3506 DMRs in the BA9 and BA24 regions were identified, respectively. Also, we compared the differences between SC and BP, and found relatively small numbers of DMRs (398 and 443) in the BA9 and BA24, respectively.

In addition, we attempted to directly compare the differences of differentially expressed genes between SC and BP in the BA9 and BA24 regions using Cuffdiff algorithm (FDR<0.05) with default options. Interestingly, a total of 0 and 34 differentially expressed genes were found between SC and BP in the BA9 and BA24 regions, respectively. These subtle expression differences between SC and BP are consistent with similar gene expression pattern between SC and BP [45].

### Brain region-specific DNA methylation linking SC with BP

**Figure 5:**
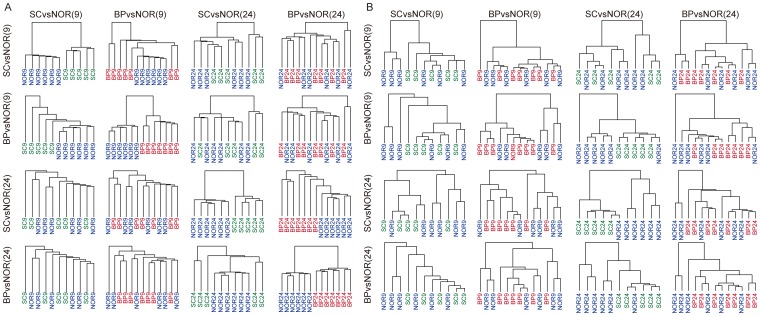
Cross cluster analyses. In a specific brain region of a given disorder, the DMRs (A) and differentially expressed genes (B) were used to distinguish patients (from the other disease or the other brain region) from normal subjects based on hierarchical clustering. Each hierarchical clustering tree described whether disease-specific DMRs (or differentially expressed genes) identified in a specific brain region, such as DMRs identified in SC vs. normal in BA9, can be used to distinguish patients (SC or BP) from normal samples in the same or distinct brain regions.

We extracted the top 50 methylation sites and the top 50 expressed genes that were most variable between case (BP or SC) and control, and generated clustering figures using these top 50 methylation sites and genes. We found that the top 50 methylation sites could explicitly distinguish disease samples from controls when compared to the top 50 expressed genes (Figure S7). Then, we sought to examine whether DMRs identified in a specific brain region of SC or BP could be used to distinguish disease patients (SC or BP) from controls in the same or distinct brain regions. We used DMRs identified in different brain regions of SC (or BP) to cluster SC vs. normal in BA9 (that is, distinguishing SC patients from controls in the BA9), SC vs. normal in BA24, BP vs. normal in BA9, and BP vs. normal in BA24. Using DMRs identified in the BA9 region of SC, we calculated normalized DNA methylation levels in the same region of BP and normal samples and performed clustering analysis. Interestingly, we found that these DMRs can successfully distinguish BP patients from normal samples (Figure 5A). However, using the DMRs identified in the BA9 region of SC could not distinguish patients (BP and SC) from normal samples based their DNA methylation levels in the BA24 region. Likewise, the DMRs identified in the BA9 region of BP can also distinguish SC from normal samples in the same region but not in the BA24 region. Similarly, DMRs in the BA24 region can distinguish both the disorders from normal subjects in the BA24 region rather than the BA9 region. Similarly, we examined whether gene expression profiles show similar tendencies. We obtained differentially expressed genes with FC>1.5 in a specific brain region of SC or BP subjects and re-calculated expression levels in case and control in two brain regions. We found that differentially expressed genes identified in a specific brain region of SC or BP subjects could not distinguish disease from normal samples, even in the same brain region (Figure 5B). In addition, we also identified differentially expressed genes using Cuffdiff with FDR<0.05. Clustering analysis exhibited consistent results (Figure S8). Taken together, our results showed that DNA methylation alterations were more stable than gene expression changes, suggesting brain region-specific DMRs might be effectively used for disease diagnosis and treatment of SC and BP.

## Discussion

Previous evidence has shown distinct DNA methylation levels in different regions of normal brain [46], [47]. Interestingly, our results demonstrated that DNA methylation alternations in SC and BP relative to normal subjects depend strongly on distinct brain regions. In the BA9 region, both SC and BP subjects showed more hypomethylated DMRs. In contrast, in the BA24 region, more hypermethylated DMRs were found. One possible explanation for the opposite patterns of DNA methylation alternations is cellular heterogeneity among different brain regions [48]. Previous studies have determined different morphologies in these two brain regions associated with SC and BP. In the BA9 region, decreased neuronal and glial density was associated with BP and elevated neuronal density was found to be associated with SC [49]. In the BA24, Öngüret al. [50] found a reduction of glia in BP subjects. It should also be noted that only a few DMRs commonly occur in the two brain regions of either SC or BP. However, relatively more overlapping DMRs between SC and BP within the same brain region were observed. The findings suggest that these common epigenetic abnormalities between SC and BP may contribute to the similar cognitive and neurobiological deficits associated with these disorders [11].

In parallel, transcriptome analyses also found a number of genes showing unique differential expression for a specific brain region of SC or BP patients, consistent with previous findings that substantial gene expression differences were observed among different regions of healthy human and mouse brains [51], [52]. In the BA9 region, we observed more up-regulated genes related with SC, yet more down-regulated genes in BP subjects. In the BA24 region, both SC and BP harbor similar numbers of up- and down-regulated genes. We further investigated the correlation (i.e. Pearson correlation coefficients using the ‘cor.test’ function in R) between DNA methylation changes of DMRs and expression changes of genes categorized by different elements (i.e. promoter, exon, intron, 5′UTR and 3′UTR) overlapping with DMRs (Figure S9). We found that expression changes of genes in which introns overlap with DMRs showed a weak but significantly positive correlation with DNA methylation changes of corresponding DMRs in the BA9 and BA24 of SC (Pearson correlation coefficient = 0.056 with p value = 0.033 and Pearson correlation coefficient = 0.073 with p value = 0.033, respectively). An inverse correlation in promoter was observed in the BA24 of BP (Pearson correlation coefficient = −0.28 with p value = 0.033), whereas a positive correlation in promoter was shown in the BA9 of BP (Pearson correlation coefficient = 0.22 with p value = 0.0497). Our findings were partially consistent with previous reports, suggesting complex relations between DNA methylation and gene expression. Subsequently, we found that 31.9%, 27.7%, 23.7% and 26.6% of DMRs were located in promoter or gene body in the BA9 and BA24 regions of SC and BP, respectively. Among them, 214, 467, 103 and 269 DMRs were located near genes with at least 1.5-fold change between case and control, and 14, 0, 137 and 1 DMRs were located near genes that are differentially expressed using Cuffdiff. Such complex relations between DNA methylation and gene expression have been observed in many studies [53], [54], and the molecular mechanisms underlying the complex relations are still poorly understood. One possible reason is that DNA methylation alternations at different genomic regions (such as introns) also contribute to control of gene expression, not just promoters [35]. Only a few DMRs overlapping with promoters were observed, however, a large number of DMRs located at the introns and intergenic regions were identified, supporting previous findings that the majority of methylated CpGs were located in intragenic and intergenic regions by generation of a map of DNA methylation from human brain [55]. A recent study further demonstrated that intragenic methylation exert functions in regulating alternative promoters, which are generally used in different contexts or tissues [56]. Another possible reason is that both DNA methylation and other epigenetic modification marks (e.g. histone modification and nucleosome locations) are required to cooperatively control gene expression [57]. Extensive cross-talk between DNA methylation and histone modification has been recently characterized [30]. DNA methylation changes may be insufficient to lead to expression changes of downstream genes.

Interestingly, DNA methylation changes (hypo- or hyper- methylation) in ten genes identified in the brain of SC and BP were also confirmed in peripheral blood samples in our previous study (under review in the Translational Psychiatry), including 1q32 [58] and 22q11.22 [59] which were considered as “hot spots” for SC and BP (Table S8). Because brain tissue availability is limited and DNA methylation changes are not limited to the brain [60], global DNA methylation abnormality in blood provides an important opportunity to develop diagnostic and therapeutic biomarkers for mental diseases [61]. In summary, this study reinforces important roles of DNA methylation and brain-region specific DNA methylation alternations in SC and BP, and highlights complex relations between DNA methylation and gene expression in the disorders.

## Supporting Information

Figure S1 Distribution of reads around CGI and gene body. The upstream and downstream 2 kb regions of CGI (A) and gene body (B) were divided into 20 equal regions. CGI and gene body were divided into 40 equal regions respectively. For each region, the normalized number of reads was calculated. DNA methylation levels across the whole chromosome 17 (C) and 19 (D).
(TIF)

Figure S2 DNA methylation levels across different chromosomes. Remarkable hypo-methylation occur in the extreme ends in the BA9 regions of SC and BP relative to normal samples.
(TIF)

Figure S3 The correlation of global DNA methylation and gene expression for each intra-class category. We used log2-transformed normalized DNA methylation levels in 10 kb windows (A) and log2-transformed gene expressions (B) to calculate the Pearson correlation coefficients between different samples from each group (case or normal individuals), separately. The numbers in the lower triangular matrixes represent Pearson correlation coefficients.
(TIF)

Figure S4 Principle component analysis. The principle component analysis of (A) methylation levels of 10 kb windows and (B) gene expression levels for both case and control samples. The x-axis and y-axis represent the first principal component and the second principal component. The colors of red, blue and green show the BP, SC and normal samples, respectively. The asterisk and diamond represent the BA9 and BA24, respectively.
(TIF)

Figure S5 Functional enrichment analyses of DMR-related genes. The top 20 biological processes determined by functional enrichment analyses of genes with different regions overlapping with DMRs.
(TIF)

Figure S6 Significant motifs enriched in DMRs of BP and SC by HOMER.
(TIF)

Figure S7 Cluster analysis. The cluster analysis of (A) methylation levels of top 50 variable DMRs and (B) expression levels of top 50 variable differentially expressed genes between case (BP or SC) and control samples.
(TIF)

Figure S8 Cross-clustering analyses of differentially expressed genes identified by Cuffdiff.
(TIF)

Figure S9 The correlations between DNA methylation and gene expression changes in various gene elements. We identified DMR-related genes in various gene elements (promoter, exon, intron, 5′ UTR and 3′ UTR) and calculated Pearson correlation coefficients and their corresponding statistical p values (using the ‘cor.test’ function in R) between changes of DNA methylation of DMRs (fold change) and expression of DMR-related genes (fold change) in different brain regions of BP and SC.
(TIF)

Table S1 Clinical information of five SC, seven BP and six normal subjects detected. (f = female, m = male; NA = not available; PMI = postmortem interval).
(XLS)

Table S2 Differentially methylated and differential expressed genes. The numbers of differentially methylated regions and their associated genes and the numbers of differentially expressed genes between the cases (SC or BP) and normal subjects.
(DOC)

Table S3 The functional enrichment analysis of DMR-related genes of BP and SC. Significances were determined using hypergeometric test with FDR<0.05. The red color represents the brain-related functions.
(XLS)

Table S4 Genes with DMRs located in their different regions.
(XLS)

Table S5 Differentially expressed genes in different comparisons.
(XLS)

Table S6 The functional enrichment analysis of differentially expressed genes. Differentially expressed genes of BP (BA9 and BA24) and SC (BA9 and BA24) identified by Cuffdiff and Fold Change method, using hypergeometric test with FDR<0.05. The red color represents the brain-related functions.
(XLS)

Table S7 The functional enrichment analysis of differential expressed genes of BP and SC using GOseq. The red color represents the brain-related functions.
(XLS)

Table S8 Ten DMR-related genes confirmed in peripheral blood samples.
(XLS)

Methods S1 Samples of BP, SC and controls used for MeDIP-seq and RNA-seq.
(DOC)
