# Supplementary figures and images for: The DNA Methylome and Transcriptome of Different Brain Regions in Schizophrenia and Bipolar Disorder
Source: PLoS One. 2014 Apr 28;9(4):e95875. doi: 10.1371/journal.pone.0095875 (PMC4002434; doi:10.1371/journal.pone.0095875)

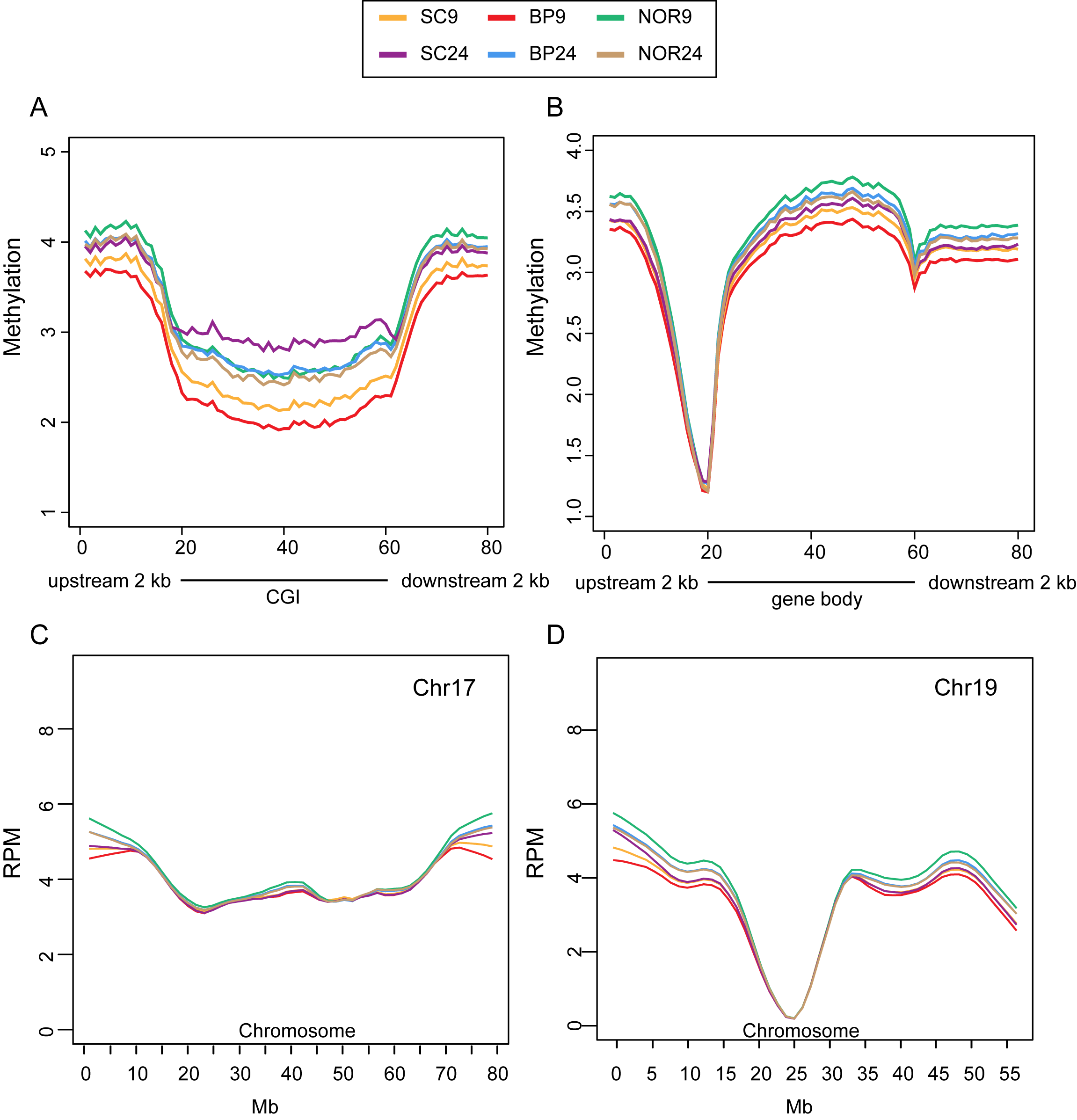

Supplement: Figure S1 — Distribution of reads around CGI and gene body. The upstream and downstream 2 kb regions of CGI (A) and gene body (B) were divided into 20 equal regions. CGI and gene body were divided into 40 equal regions respectively. For each region, the normalized number of reads was calculated. DNA methylation levels across the whole chromosome 17 (C) and 19 (D). (TIF) [file pone.0095875.s001.tif]

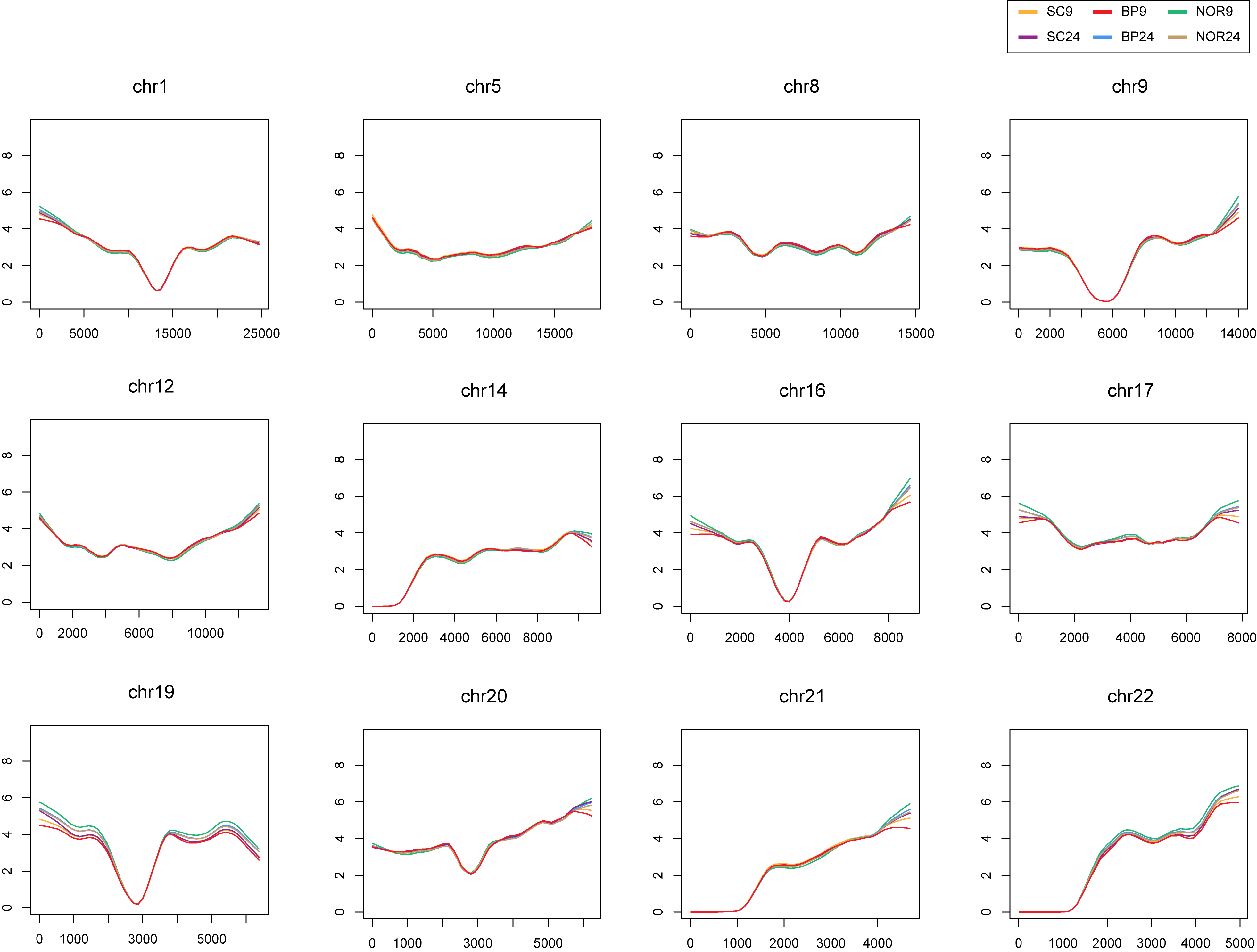

Supplement: Figure S2 — DNA methylation levels across different chromosomes. Remarkable hypo-methylation occur in the extreme ends in the BA9 regions of SC and BP relative to normal samples. (TIF) [file pone.0095875.s002.tif]

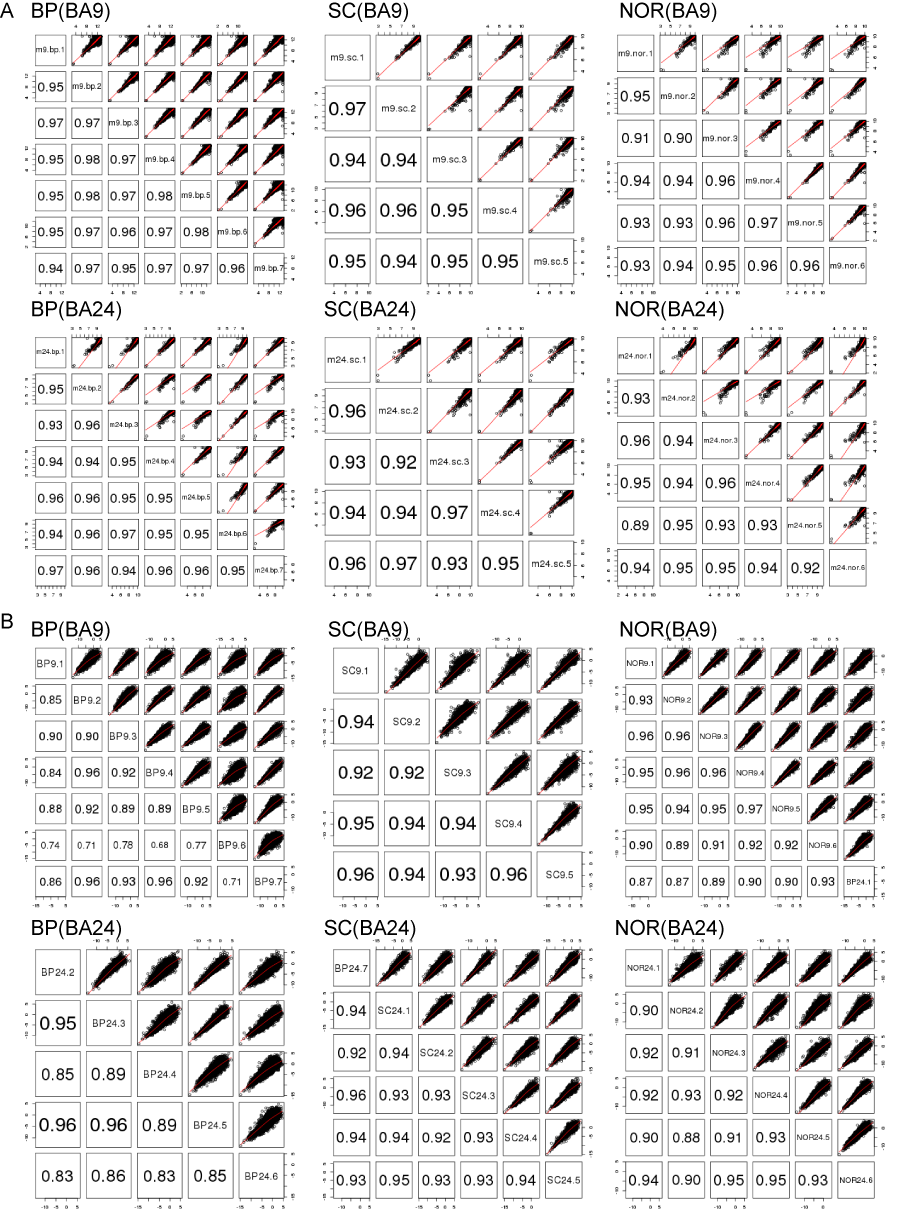

Supplement: Figure S3 — The correlation of global DNA methylation and gene expression for each intra-class category. We used log2-transformed normalized DNA methylation levels in 10 kb windows (A) and log2-transformed gene expressions (B) to calculate the Pearson correlation coefficients between different samples from each group (case or normal individuals), separately. The numbers in the lower triangular matrixes represent Pearson correlation coefficients. (TIF) [file pone.0095875.s003.tif]

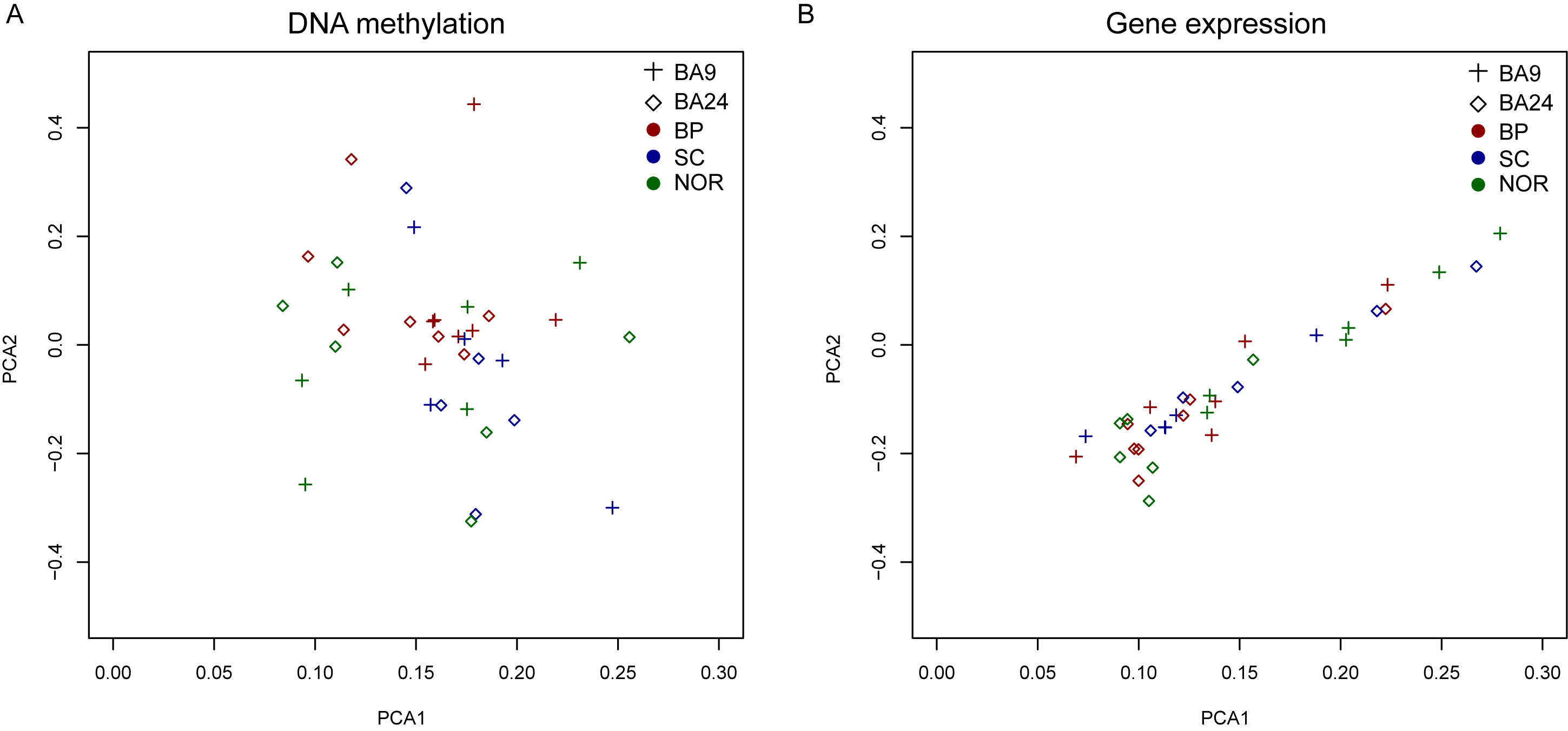

Supplement: Figure S4 — Principle component analysis. The principle component analysis of (A) methylation levels of 10 kb windows and (B) gene expression levels for both case and control samples. The x-axis and y-axis represent the first principal component and the second principal component. The colors of red, blue and green show the BP, SC and normal samples, respectively. The asterisk and diamond represent the BA9 and BA24, respectively. (TIF) [file pone.0095875.s004.tif]

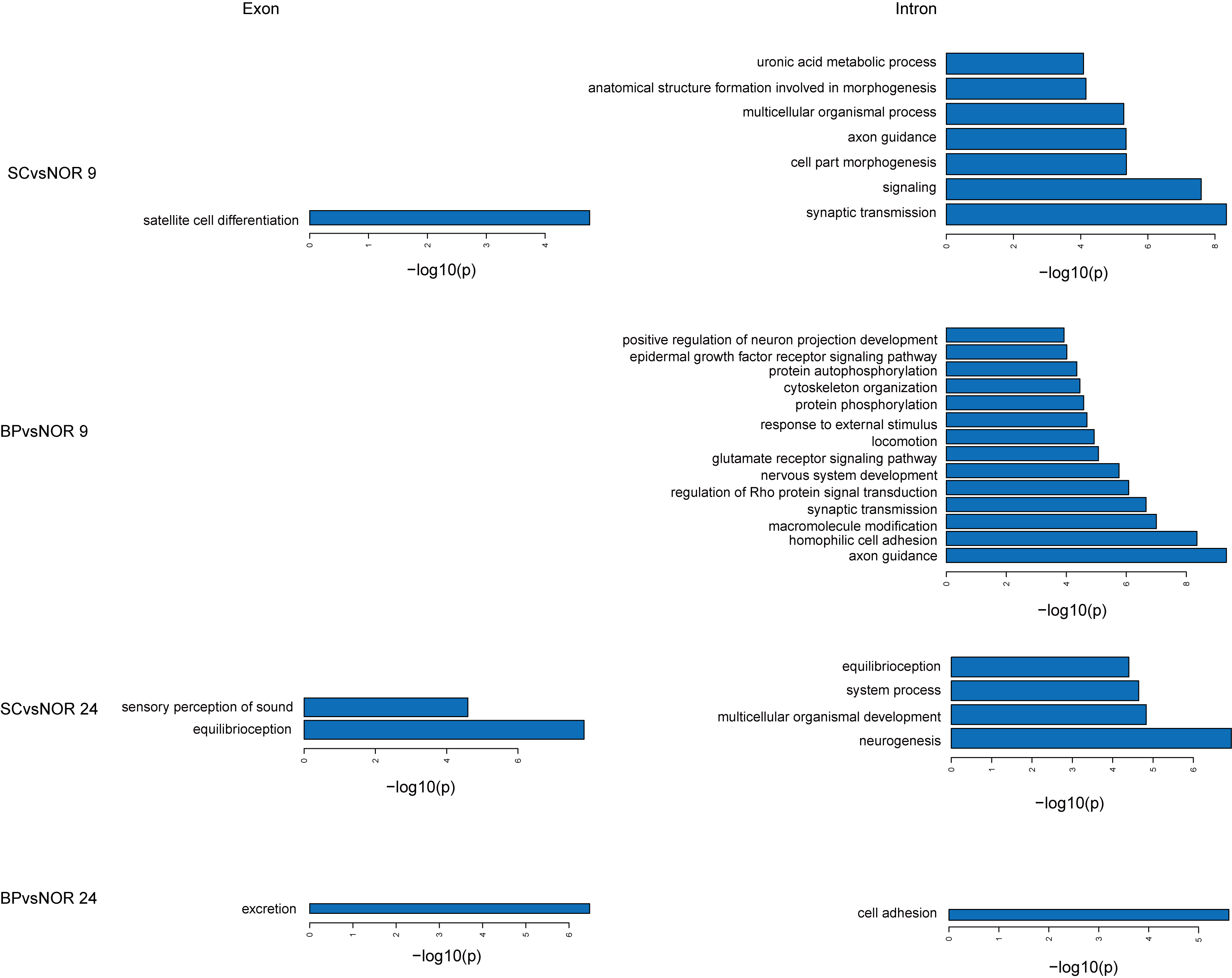

Supplement: Figure S5 — Functional enrichment analyses of DMR-related genes. The top 20 biological processes determined by functional enrichment analyses of genes with different regions overlapping with DMRs. (TIF) [file pone.0095875.s005.tif]

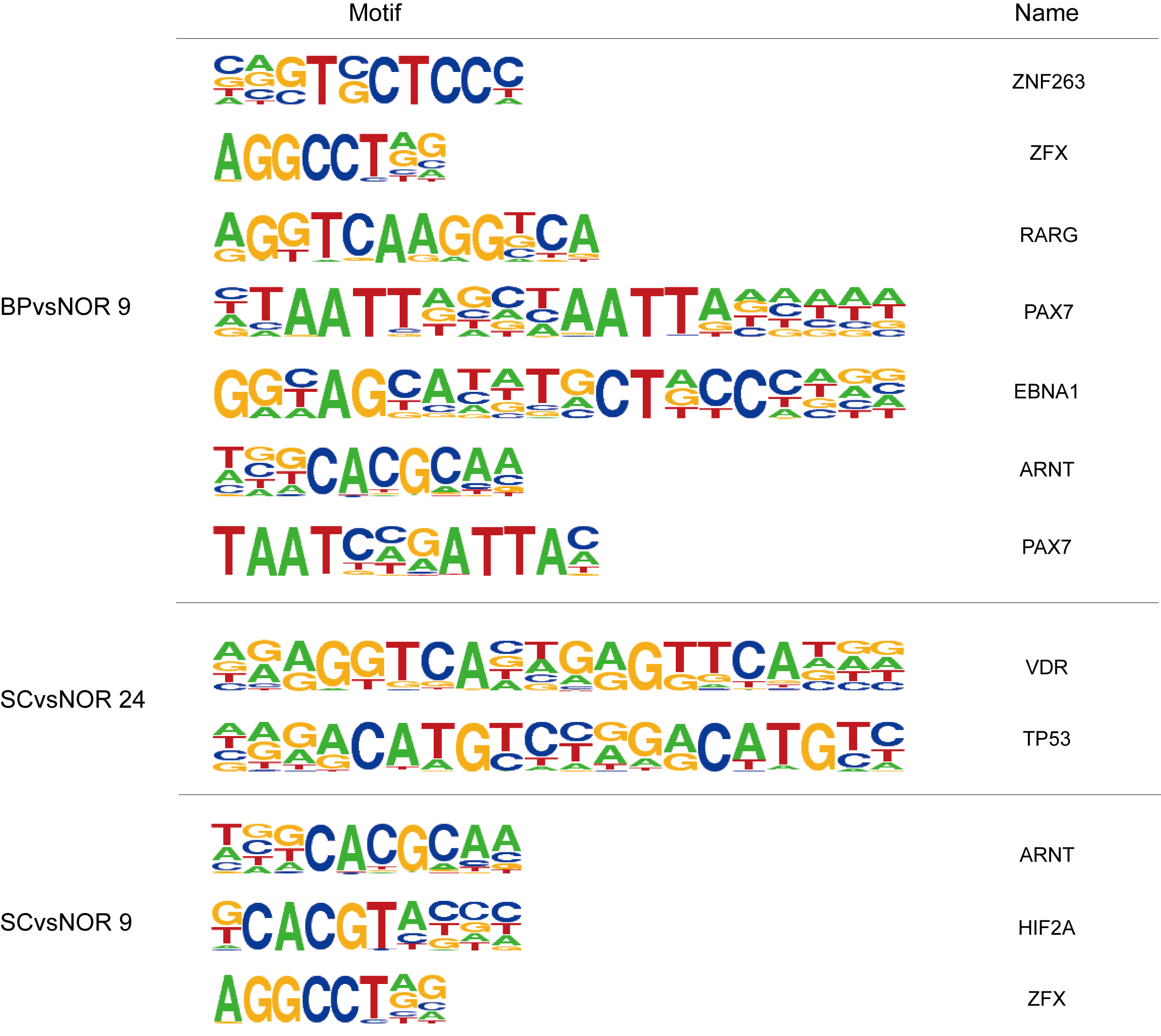

Supplement: Figure S6 — Significant motifs enriched in DMRs of BP and SC by HOMER. (TIF) [file pone.0095875.s006.tif]

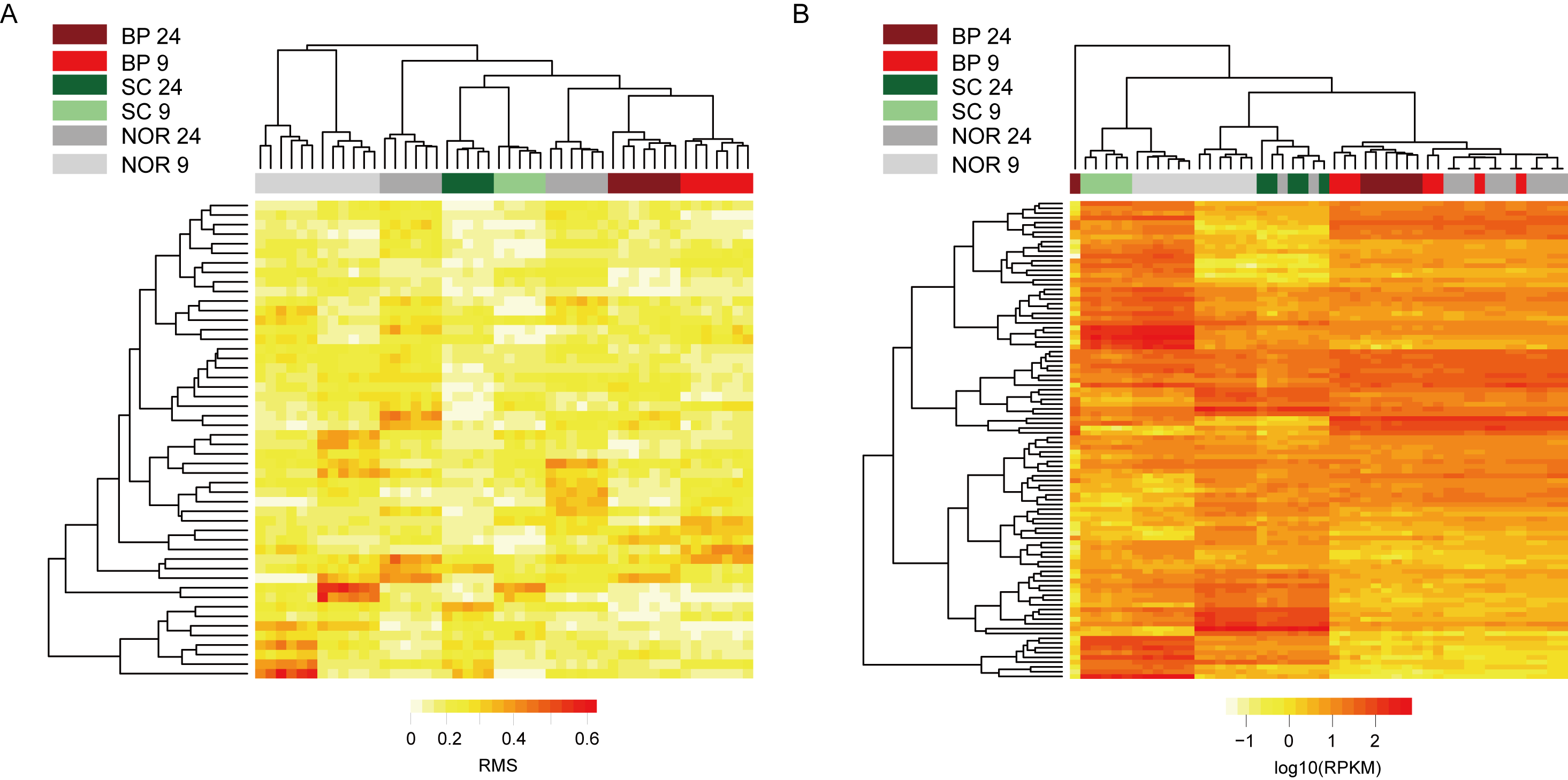

Supplement: Figure S7 — Cluster analysis. The cluster analysis of (A) methylation levels of top 50 variable DMRs and (B) expression levels of top 50 variable differentially expressed genes between case (BP or SC) and control samples. (TIF) [file pone.0095875.s007.tif]

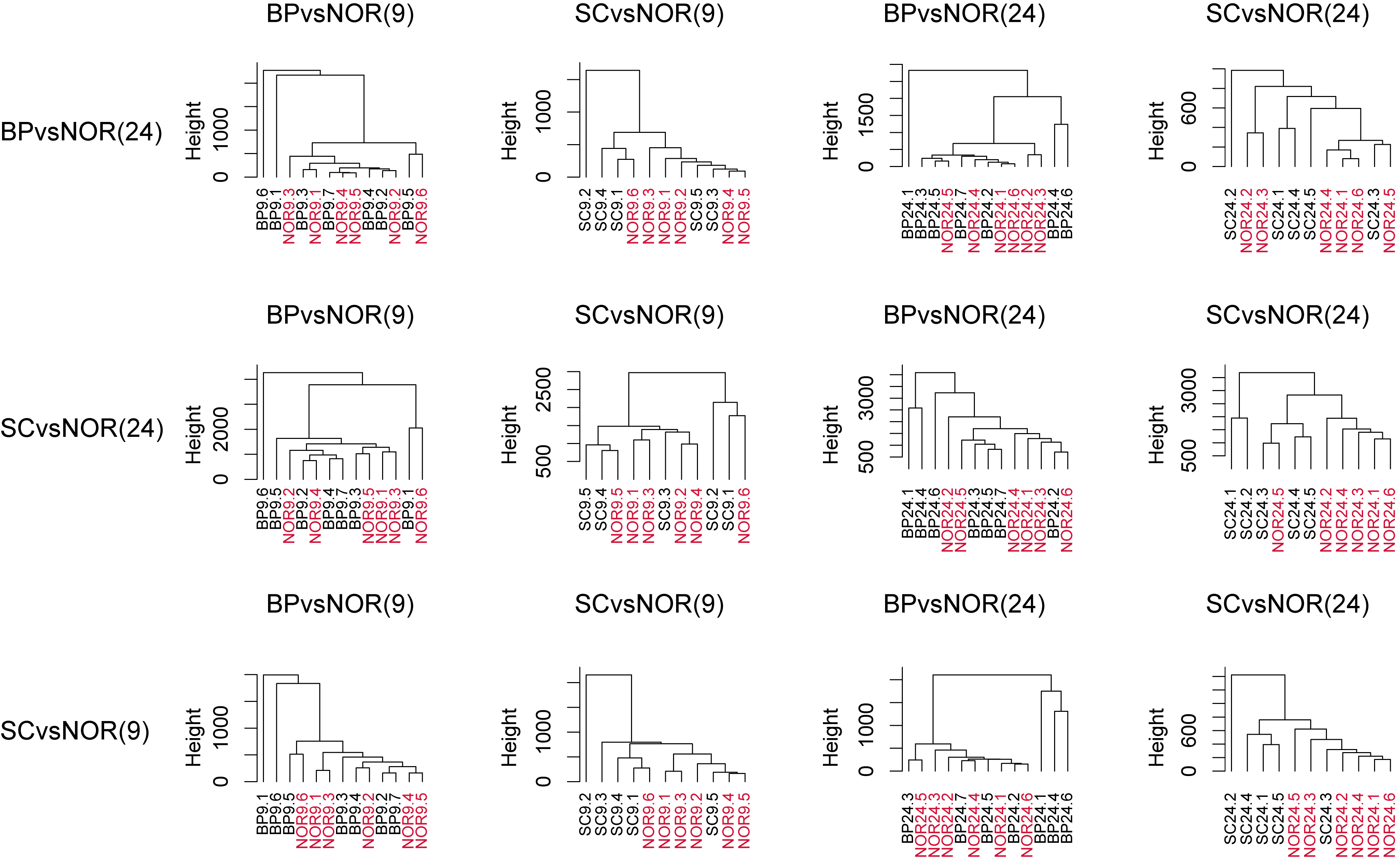

Supplement: Figure S8 — Cross-clustering analyses of differentially expressed genes identified by Cuffdiff. (TIF) [file pone.0095875.s008.tif]

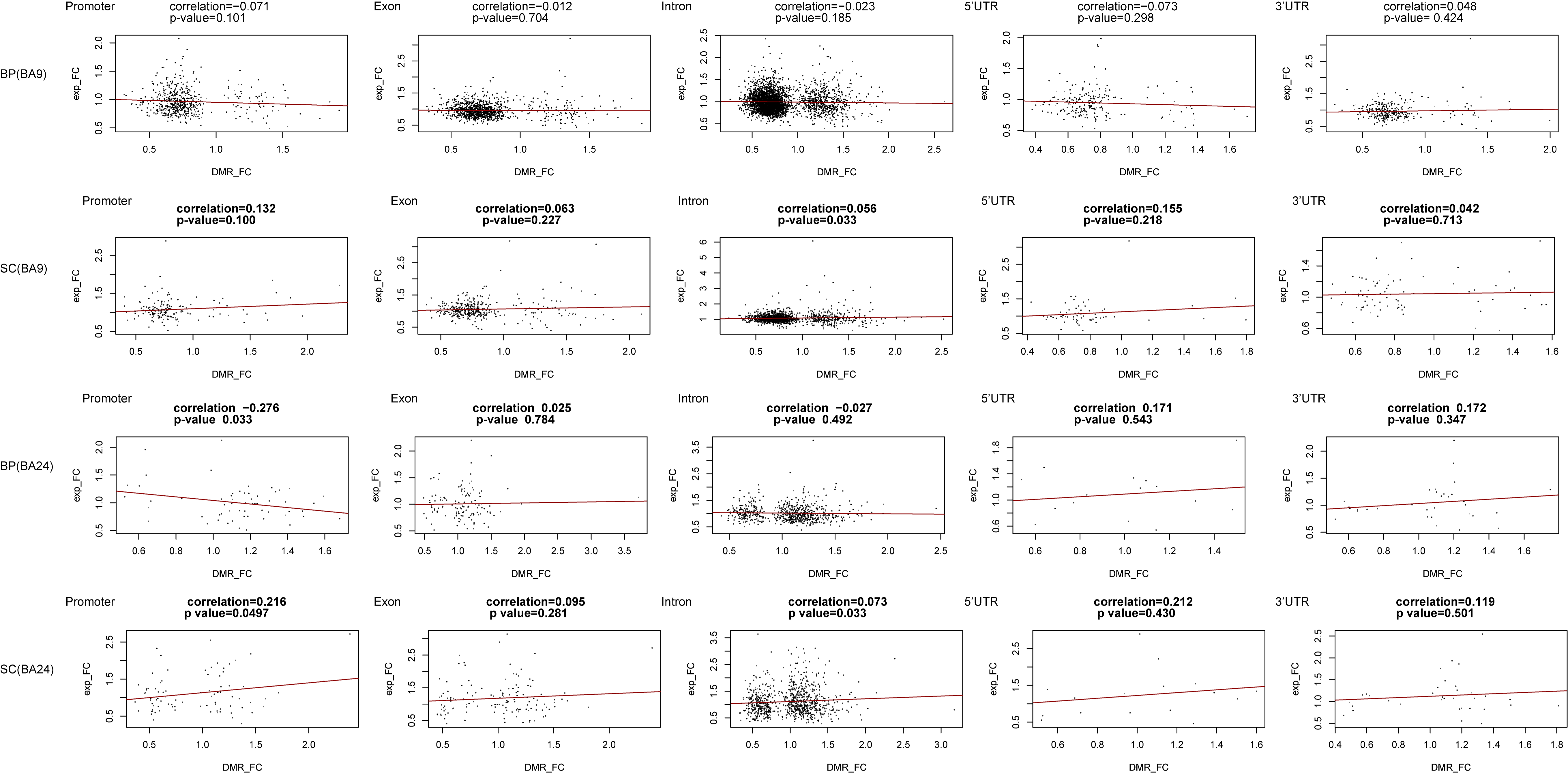

Supplement: Figure S9 — The correlations between DNA methylation and gene expression changes in various gene elements. We identified DMR-related genes in various gene elements (promoter, exon, intron, 5′ UTR and 3′ UTR) and calculated Pearson correlation coefficients and their corresponding statistical p values (using the ‘cor.test’ function in R) between changes of DNA methylation of DMRs (fold change) and expression of DMR-related genes (fold change) in different brain regions of BP and SC. (TIF) [file pone.0095875.s009.tif]
