# Supplementary material for: The DNA Methylome and Transcriptome of Different Brain Regions in Schizophrenia and Bipolar Disorder
Source: PLoS One. 2014 Apr 28;9(4):e95875. doi: 10.1371/journal.pone.0095875 (PMC4002434; doi:10.1371/journal.pone.0095875)
Supplement: Table S2 — Differentially methylated and differential expressed genes. The numbers of differentially methylated regions and their associated genes and the numbers of differentially expressed genes between the cases (SC or BP) and normal subjects. (DOC) [file pone.0095875.s011.doc]

**Supplementary Table S2.** The numbers of differentially methylated regions and their associated genes and the numbers of differentially expressed genes between the cases (SC or BP) and normal subjects

|  | DNA Methylome | | | | | | | | Transcriptome | |
| --- | --- | --- | --- | --- | --- | --- | --- | --- | --- | --- |
|  |  | Num of DMRs | Promoter | 5' UTR | Exon | Intron | 3' UTR | All |
| SC vs. NOR (BA9) | Hyper- | 1699 | 31 | 14 | 86 | 427 | 21 | 454 | Up | 761 |
|  | Hypo- | 3286 | 198 | 76 | 390 | 1452 | 77 | 1586 | Down | 316 |
| BP vs. NOR (BA9) | Hyper- | 4706 | 120 | 36 | 223 | 967 | 66 | 1051 | Up | 230 |
|  | Hypo- | 9219 | 630 | 213 | 1153 | 3410 | 288 | 3772 | Down | 1855 |
| SC vs. NOR (BA24) | Hyper- | 2890 | 102 | 35 | 192 | 795 | 44 | 863 | Up | 1871 |
|  | Hypo- | 977 | 51 | 7 | 62 | 443 | 10 | 478 | Down | 1768 |
| BP vs. NOR (BA24) | Hyper- | 2048 | 83 | 21 | 161 | 659 | 40 | 717 | Up | 772 |
|  | Hypo- | 624 | 11 | 7 | 42 | 272 | 9 | 284 | Down | 871 |
