# Supplementary material for: The DNA Methylome and Transcriptome of Different Brain Regions in Schizophrenia and Bipolar Disorder
Source: PLoS One. 2014 Apr 28;9(4):e95875. doi: 10.1371/journal.pone.0095875 (PMC4002434; doi:10.1371/journal.pone.0095875)
Supplement: Methods S1 — Samples of BP, SC and controls used for MeDIP-seq and RNA-seq. (DOC) [file pone.0095875.s018.doc]

**Methods S1**

**Subjects**

Brain tissues from control [^1^](#_ENREF_1), schizophrenia (N=5, SC) and bipolar disorder (N=7, BP) subjects were obtained from the Southwest Brain Bank (SWBB) with consent from the next-of-kin (NOK). The SWBB collection of postmortem tissue for research was conducted under the jurisdiction of the State of Texas Anatomical Review Board. All interviews with the NOK have been determined to be exempt from ethical review by the The University of Texas Health Science Center at San Antonio (UTHSCSA) IRB. The NOK agreed to provide the donation and they read a State approved form. We telephoned the NOK and recorded their agreement to donate. The NOK interview (psychological autopsy) about the donor was performed by trained clinicians. To establish the clinical diagnosis a DSM-IV based Mini-International Neuropsychiatric Interview (M.I.N.I.)[^2^](#_ENREF_2) was administrated to the NOK about the deceased. This information in addition to all medical records that were obtained was presented to the expert diagnostician consensus group, whose inter-rater reliability for the MINI was 0.8 for SC and BP. Medications listed were those that were prescribed at the time of death. In order to estimate psychotic symptoms in the subjects at the time of death in the NOK interview a retrospective Bipolar Inventory of Symptoms Scale (BISS) was administrated. The BISS generated a total severity score and 5 symptom factor scores: mania, depression, anxiety, irritability and psychosis[^3^](#_ENREF_3). The psychosis factor composed of the questions about paranoid delusions, hallucinations and impaired insight was used to estimate the presence of psychosis in the last week of life.

Subject characteristics were listed in Supplementary Table S1A. Groups were matched for age, sex, PMI, pH, and age of illness onset for the affected groups (Supplementary Table S1B). There were three suicides in the BP group. There was a no statistical difference between BISS Psychosis factor scores across diagnoses (Mann Whitney-U test, p>0.05).

**Postmortem tissue**

The cerebrum was hemisected and cut into 1 cm-thick coronal blocks starting at the frontal pole, digitally photographed to document anatomical location, immediately frozen in isopentane (2-methylbutane, Fisher), chilled with dry ice to -60°C, and then stored at -80°C. Tissue quality was determined by a neuro-pathologist through both gross and microscopic neuropathological examinations. All subjects included in this study were free of confounding neuropathology. Toxicology results were obtained from the Bexar County Forensic Toxicology Laboratory via the autopsy report. Control tissue was toxicology free of neurotropic medications (supplementary Table S1). For tissue identification of Brodmann areas 9 and 24, we used the criteria described by Rajkowska and Goldman-Rakic [^4^](#_ENREF_4) and taken from the same hemisphere.

**DNA and RNA preparation**

All of our samples were from fresh-frozen specimens that were stored in -80°C freezers. Tissue storage time was 1-3 years. The samples were collected from the larger blocks containing the appropriate brain region and RNA/DNA was isolated within a few days of dissection from the larger blocks of tissue. These samples were from postmortem cases with SC and BP who had a postmortem interval (PMI) (the time of death until the time of tissue preservation) of about 24 hours. As a result, RNA integrity (RIN) was lower compared to animal models or in vitro studies.

**References**

1. Del Vecchio A, Latini G, Henry E, Christensen RD. Template bleeding times of 240 neonates born at 24 to 41 weeks gestation. J Perinatol 2008; 28:427-31.

2. Sheehan DV, Lecrubier Y, Sheehan KH, Amorim P, Janavs J, Weiller E, Hergueta T, Baker R, Dunbar GC. The Mini-International Neuropsychiatric Interview (M.I.N.I.): the development and validation of a structured diagnostic psychiatric interview for DSM-IV and ICD-10. The Journal of clinical psychiatry 1998; 59 Suppl 20:22-33;quiz 4-57.

3. Thompson PM, Gonzalez JM, Singh V, Schoolfield JD, Katz MM, Bowden CL. Principal domains of behavioral psychopathology identified by the Bipolar Inventory of Signs and Symptoms Scale (BISS). Psychiatry research 2010; 175:221-6.

4. Rajkowska G, Goldman-Rakic PS. Cytoarchitectonic definition of prefrontal areas in the normal human cortex: II. Variability in locations of areas 9 and 46 and relationship to the Talairach Coordinate System. Cerebral cortex 1995; 5:323-37.
